# Creatininium bis­(pyridine-2,6-dicarboxyl­ato)chromate(III) pyridine-2,6-dicarboxylic acid hexa­hydrate

**DOI:** 10.1107/S1600536808027700

**Published:** 2008-09-06

**Authors:** Hossein Aghabozorg, Zohreh Derikvand, Marilyn M. Olmstead, Jafar Attar Gharamaleki

**Affiliations:** a Faculty of Chemistry, Tarbiat Moallem University, Tehran, Iran; b Department of Chemistry, University of California, One Shields Avenue, Davis, CA 95616-5292, USA

## Abstract

The title compound, (C_4_H_8_N_3_O)[Cr(C_7_H_3_NO_4_)_2_]·C_7_H_5_NO_4_·6H_2_O, was obtained by the reaction of Cr(NO_3_)_3_·9H_2_O with pyridine-2,6-dicarboxylic acid (pydcH_2_) and creatinine (creat) in aqueous solution (molar ratio 1:2:2). The cation is a protonated creatinine (creatH^+^) while the anion is a bis-pydc^2−^ Cr^III^ complex. The Cr^III^ is coordinated by four oxygen and two nitro­gen atoms of two (pydc)^2–^ groups and has a disorted octa­hedral coordination environment. The structure also contains a neutral mol­ecule of pydcH_2_ that is hydrogen bonded to the creatH^+^ and six mol­ecules of water. Extensive inter­molecular inter­actions, including seventeen classical hydrogen bonds, two weak C—H⋯O bonds, and C—O⋯π stacking inter­actions, with O⋯centroid distances of 3.211 (13) and 3.300 (12) Å, connect the various components in the crystal structure.

## Related literature

For a recent review on proton-transfer compounds and their structures, see: Aghabozorg, Manteghi *et al.* (2008 ▶). For related creatininium structures, see: Aghabozorg, Ramezanipour *et al.* (2008 ▶); Moghimi *et al.* (2004 ▶, 2005 ▶).
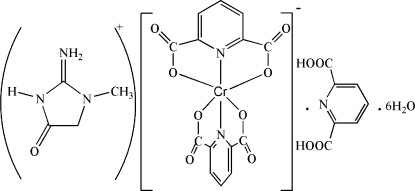


## Experimental

### 

#### Crystal data

(C_4_H_8_N_3_O)[Cr(C_7_H_3_NO_4_)_2_]·C_7_H_5_NO_4_·6H_2_O
*M* *_r_* = 771.56
Triclinic, 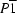

*a* = 9.0860 (5) Å
*b* = 13.6274 (8) Å
*c* = 14.7301 (8) Å
α = 65.481 (2)°
β = 74.685 (2)°
γ = 77.644 (2)°
*V* = 1589.10 (15) Å^3^
*Z* = 2
Mo *K*α radiation
μ = 0.46 mm^−1^
*T* = 90 (2) K
0.33 × 0.32 × 0.10 mm

#### Data collection

Bruker SMART APEXII diffractometer
Absorption correction: multi-scan (*SADABS*; Sheldrick, 1996 ▶) *T*_min_ = 0.864, *T*_max_ = 0.956
20278 measured reflections
7266 independent reflections
6654 reflections with *I* > 2σ(*I*)
*R*_int_ = 0.019

#### Refinement

*R*[*F* ^2^ > 2σ(*F* ^2^)] = 0.028
*wR*(*F* ^2^) = 0.077
*S* = 1.01
7266 reflections
584 parameters
All H-atom parameters refined
Δρ_max_ = 0.41 e Å^−3^
Δρ_min_ = −0.46 e Å^−3^

### 

Data collection: *APEX2* (Bruker, 2007 ▶); cell refinement: *SAINT* (Bruker, 2007 ▶); data reduction: *SAINT*; program(s) used to solve structure: *SHELXS97* (Sheldrick, 2008 ▶); program(s) used to refine structure: *SHELXL97* (Sheldrick, 2008 ▶); molecular graphics: *SHELXTL* (Sheldrick, 2008 ▶); software used to prepare material for publication: *SHELXL97*.

## Supplementary Material

Crystal structure: contains datablocks I, global. DOI: 10.1107/S1600536808027700/sj2533sup1.cif

Structure factors: contains datablocks I. DOI: 10.1107/S1600536808027700/sj2533Isup2.hkl

Additional supplementary materials:  crystallographic information; 3D view; checkCIF report

## Figures and Tables

**Table:** 

Cr1—N1	1.9733 (11)
Cr1—N2	1.9769 (11)
Cr1—O5	1.9842 (9)
Cr1—O3	1.9942 (10)
Cr1—O1	1.9947 (9)
Cr1—O7	1.9974 (9)

**Table:** 

N1—Cr1—N2	172.88 (4)
N1—Cr1—O5	106.16 (4)
N2—Cr1—O5	79.39 (4)
N1—Cr1—O3	78.84 (4)
N2—Cr1—O3	96.78 (4)
O5—Cr1—O3	91.39 (4)
N1—Cr1—O1	79.13 (4)
N2—Cr1—O1	105.30 (4)
O5—Cr1—O1	93.27 (4)
O3—Cr1—O1	157.91 (4)
N1—Cr1—O7	96.08 (4)
N2—Cr1—O7	78.42 (4)
O5—Cr1—O7	157.76 (4)
O3—Cr1—O7	92.80 (4)

**Table 2:** Hydrogen-bond geometry (Å, °)

*D*—H⋯*A*	*D*—H	H⋯*A*	*D*⋯*A*	*D*—H⋯*A*
N4—H4*A*⋯O11	0.83 (2)	2.08 (2)	2.8934 (16)	167.3 (19)
N4—H4*B*⋯O9	0.85 (2)	1.98 (2)	2.8343 (16)	178.5 (8)
O10—H10*A*⋯O15	0.91 (2)	1.63 (2)	2.5382 (15)	176 (2)
O14—H14*B*⋯O8	0.83 (2)	2.01 (2)	2.8255 (16)	169 (2)
O15—H15*A*⋯O14	0.86 (2)	1.82 (2)	2.6718 (17)	168 (2)
O16—H16*A*⋯O4	0.78 (2)	2.07 (2)	2.8006 (15)	156 (2)
O17—H17*B*⋯O3	0.79 (2)	1.99 (2)	2.7758 (14)	172 (2)
O18—H18*A*⋯O2	0.84 (2)	1.98 (2)	2.7927 (15)	162 (2)
O19—H19*A*⋯O6	0.85 (3)	1.95 (3)	2.7763 (15)	164 (2)
N5—H5*A*⋯O16^i^	0.85 (2)	1.87 (2)	2.7175 (16)	175 (2)
O12—H12*A*⋯O17^ii^	0.91 (2)	1.66 (3)	2.5720 (14)	176 (2)
O14—H14*A*⋯O18^iii^	0.79 (3)	1.98 (3)	2.7546 (17)	167 (2)
O15—H15*B*⋯O7^iii^	0.77 (2)	2.16 (2)	2.9105 (15)	162 (2)
O16—H16*B*⋯O9^iv^	0.83 (3)	2.05 (3)	2.8083 (15)	152 (2)
O17—H17*A*⋯O19^v^	0.85 (2)	1.84 (2)	2.6916 (16)	178 (2)
O18—H18*B*⋯O8^vi^	0.84 (3)	2.12 (3)	2.9558 (15)	171 (2)
O19—H19*B*⋯O13^vii^	0.81 (2)	2.17 (2)	2.9531 (16)	162 (2)
C5—H5⋯O7^viii^	0.94 (2)	2.392 (19)	3.251 (2)	152.2 (15)
C10—H10⋯O6^ii^	0.929 (19)	2.283 (19)	3.0919 (17)	145.4 (18)

Symmetry codes: (i) 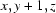; (ii) 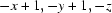; (iii) 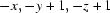; (iv) 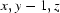; (v) 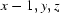; (vi) 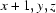; (vii) 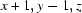; (viii) 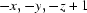.

**Fig. 1.**
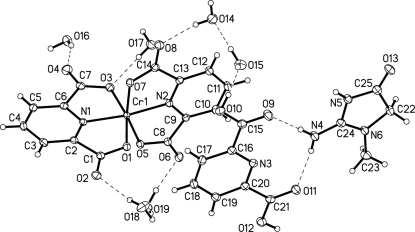
A view of the asymmetric unit of the title compound with the atom numbering scheme. Displacement ellipsoids are drawn at the 50% probability level. Hydrogen bonds are shown as dashed lines.

**Fig. 2.**
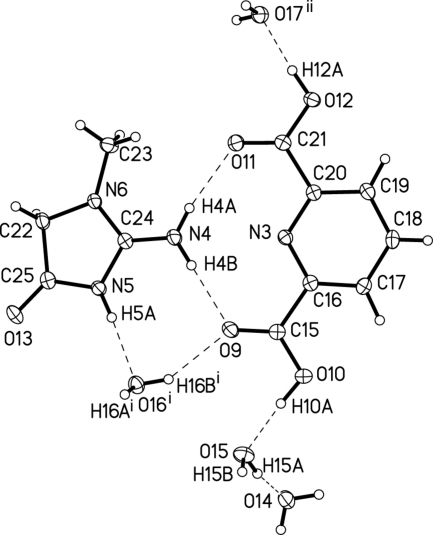
Details of the hydrogen bonding between the creatininium cation, the neutral pydcH2 molecule and the uncoordinated water molecules. Symmetry codes: (i) x, 1 + y, z, (ii) 1 - x, 1 - y, -z.

**Fig. 3.**
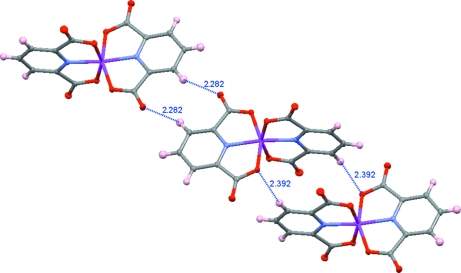
One dimensional chains of [Cr(pydc)2]- anions that are generated by C–H···O hydrogen bonds.

**Fig. 4.**
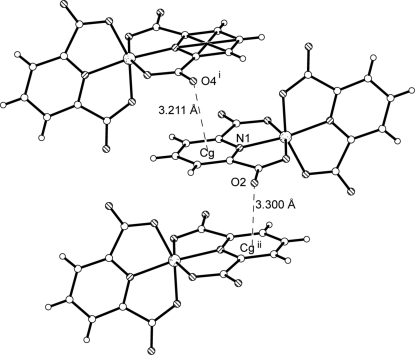
C–O···π stacking interactions in the title compound. Distances are reported between O atoms and the centroid (Cg) of the N1/C2-C6 aromatic ring. Symmetry codes: (i) 1-x, -y, 1-z), (ii) (-x, -y, 1-z).

## supplementary crystallographic information

### Comment

We have previously reported several structures that contain creatinine, pyridine-2,6-dicarboxylic acid and various metals such as: (creatH)(pydcH)^.^H_2_O (Moghimi *et al.*, 2004), (creatH)_2_[Bi(pydc)_2_]_2_^.^4H_2_O (Moghimi *et al.*, 2005) and (creatH)[Zn(pydc)(pydcH)]^.^ 4H_2_O (Aghabozorg, Ramezanipour *et al.*, 2008). For more details and related literature see our recent review article (Aghabozorg, Manteghi *et al.*, 2008).

The asymmetric unit  of the title compound Fig. 1, contains a [Cr(pydc)_2_]^-^ anion, a (creatH)^+^ cation,  a pydcH_2_ molecule and six uncoordinated water molecules. In the anions, Cr^III^ has a N_2_O_4_ donor set with normal distances and angles (Table 1). The two (pydc)^2–^ planes form a dihedral angle of 89.64 (1)°, and thus are virtually perpendicular to each other.

As depicted in Fig. 2, the creatininium ion is evidently strongly associated with the neutral molecule of pydcH_2_ through two N—H···O hydrogen bonds and the two units are bridged by one of the water molecules.  There are also two rather strong hydrogen bonds between the two carboxylic acid OH groups of the pydcH_2_ and molecules of water. The relevant O···O distances are 2.5382 (15) and 2.5720 (14)Å. This structural arrangement between cation and neutral pydcH_2_ markedly differs from the arrangement in the ion pair creatH^+^pydcH^-^ previously reported in the structure of the creatH^+^pydcH^-^ monohydrate (Moghimi *et al.*, 2004). In the latter structure, only one intramolecular hydrogen bond is formed between one of the NH_2_ H atoms and the carboxylate group. The other NH_2_ H atom is hydrogen bonded to the molecule of water.

In addition to numerous strong hydrogen bonds, intermolecular interactions in the the title compound include weaker C—H···O interactions which link the anions together, shown in Fig. 3 and Table 2, as well as C—O···π stacking interactions between CO groups of carboxylate fragments and aromatic rings of (pydc)^2–^ with O···centroid distances of 3.211 (13) and 3.300 (12) Å (Fig. 4).

### Experimental

The reaction between pyridine-2,6-dicarboxylic acid (100 mg, 1 mmol) in 10 ml water, creatinine (creat) (110 mg, 1 mmol) in 20 ml water and chromium(III) nitrate nonahydrate (100 mg, 0.5 mmol) in 5 ml water at 2:2:1 molar ratio gave violet crystals after slow evaporation of the solvent at the room temperature.

### Refinement

All H atoms were freely refined with isotropic thermal parameters.

### Crystal data

**Table:**

(C_4_H_8_N_3_O)[Cr(C_7_H_3_NO_4_)_2_]·C_7_H_5_NO_4_·6H_2_O	*Z* = 2
*M**_r_* = 771.56	*F*(000) = 798
Triclinic, *P*1	*D*_x_ = 1.612 Mg m^−^^3^
Hall symbol: -P 1	Mo *K*α radiation, λ = 0.71073 Å
*a* = 9.0860 (5) Å	Cell parameters from 6379 reflections
*b* = 13.6274 (8) Å	θ = 3.0–31.5°
*c* = 14.7301 (8) Å	µ = 0.46 mm^−^^1^
α = 65.481 (2)°	*T* = 90 K
β = 74.685 (2)°	Plate, violet
γ = 77.644 (2)°	0.33 × 0.32 × 0.10 mm
*V* = 1589.10 (15) Å^3^	

### Data collection

**Table:**

Bruker SMART APEXII diffractometer	7266 independent reflections
Radiation source: fine-focus sealed tube	6654 reflections with *I* > 2σ(*I*)
graphite	*R*_int_ = 0.019
Detector resolution: 8.3 pixels mm^-1^	θ_max_ = 27.5°, θ_min_ = 2.7°
ω scans	*h* = −11→11
Absorption correction: multi-scan (SADABS; Sheldrick, 1996)	*k* = −17→17
*T*_min_ = 0.864, *T*_max_ = 0.956	*l* = −19→19
20278 measured reflections	

### Refinement

**Table:**

Refinement on *F*^2^	Primary atom site location: structure-invariant direct methods
Least-squares matrix: full	Secondary atom site location: difference Fourier map
*R*[*F*^2^ > 2σ(*F*^2^)] = 0.028	Hydrogen site location: inferred from neighbouring sites
*wR*(*F*^2^) = 0.077	All H-atom parameters refined
*S* = 1.02	*w* = 1/[σ^2^(*F*_o_^2^) + (0.0385*P*)^2^ + 0.9325*P*] where *P* = (*F*_o_^2^ + 2*F*_c_^2^)/3
7266 reflections	(Δ/σ)_max_ = 0.001
584 parameters	Δρ_max_ = 0.41 e Å^−^^3^
0 restraints	Δρ_min_ = −0.46 e Å^−^^3^

### Special details

**Table:**

Geometry. All e.s.d.'s (except the e.s.d. in the dihedral angle between two l.s. planes) are estimated using the full covariance matrix. The cell e.s.d.'s are taken into account individually in the estimation of e.s.d.'s in distances, angles and torsion angles; correlations between e.s.d.'s in cell parameters are only used when they are defined by crystal symmetry. An approximate (isotropic) treatment of cell e.s.d.'s is used for estimating e.s.d.'s involving l.s. planes.
Refinement. Refinement of *F*^2^ against ALL reflections. The weighted *R*-factor *wR* and goodness of fit *S* are based on *F*^2^, conventional *R*-factors *R* are based on *F*, with *F* set to zero for negative *F*^2^. The threshold expression of *F*^2^ > σ(*F*^2^) is used only for calculating *R*-factors(gt) *etc*. and is not relevant to the choice of reflections for refinement. *R*-factors based on *F*^2^ are statistically about twice as large as those based on *F*, and *R*- factors based on ALL data will be even larger.

### Fractional atomic coordinates and isotropic or equivalent isotropic displacement parameters (Å^2^)

**Table:**

	*x*	*y*	*z*	*U*_iso_*/*U*_eq_	
Cr1	0.25265 (2)	0.216209 (16)	0.359634 (15)	0.01061 (6)	
O1	0.36533 (10)	0.23292 (7)	0.45071 (7)	0.01405 (18)	
O2	0.46030 (11)	0.14296 (8)	0.59416 (7)	0.0175 (2)	
O3	0.15065 (11)	0.14097 (8)	0.30655 (7)	0.01496 (19)	
O4	0.07696 (12)	−0.01935 (8)	0.33737 (8)	0.0200 (2)	
O5	0.43892 (10)	0.22188 (7)	0.25150 (7)	0.01432 (19)	
O6	0.55182 (11)	0.32381 (8)	0.09398 (7)	0.0181 (2)	
O7	0.05282 (10)	0.26969 (7)	0.43082 (7)	0.01383 (18)	
O8	−0.14469 (11)	0.40222 (8)	0.41140 (8)	0.0189 (2)	
N1	0.26631 (12)	0.06706 (9)	0.46327 (8)	0.0116 (2)	
N2	0.21136 (12)	0.36451 (9)	0.25887 (8)	0.0120 (2)	
C1	0.38970 (14)	0.14811 (10)	0.53165 (10)	0.0130 (2)	
C2	0.32378 (14)	0.04896 (10)	0.54427 (10)	0.0126 (2)	
C3	0.31731 (15)	−0.05060 (11)	0.62527 (10)	0.0154 (3)	
H3	0.3540 (19)	−0.0619 (13)	0.6818 (13)	0.016 (4)*	
C4	0.25291 (15)	−0.13028 (11)	0.61755 (10)	0.0166 (3)	
H4	0.2463 (19)	−0.1981 (14)	0.6707 (13)	0.017 (4)*	
C5	0.19581 (15)	−0.11032 (11)	0.53165 (11)	0.0156 (3)	
H5	0.150 (2)	−0.1614 (14)	0.5244 (13)	0.020 (4)*	
C6	0.20238 (14)	−0.00750 (10)	0.45504 (10)	0.0131 (2)	
C7	0.13731 (14)	0.03755 (11)	0.35899 (10)	0.0140 (2)	
C8	0.44766 (15)	0.30926 (10)	0.16929 (10)	0.0133 (2)	
C9	0.31394 (14)	0.39718 (10)	0.17162 (10)	0.0128 (2)	
C10	0.28865 (16)	0.49990 (11)	0.09810 (10)	0.0154 (3)	
H10	0.357 (2)	0.5255 (15)	0.0365 (14)	0.023 (4)*	
C11	0.15425 (16)	0.56585 (11)	0.11809 (10)	0.0164 (3)	
H11	0.135 (2)	0.6360 (15)	0.0687 (14)	0.023 (4)*	
C12	0.04727 (15)	0.52912 (11)	0.20890 (10)	0.0152 (3)	
H12	−0.0436 (19)	0.5732 (14)	0.2231 (12)	0.015 (4)*	
C13	0.08062 (14)	0.42558 (10)	0.27889 (10)	0.0127 (2)	
C14	−0.01527 (15)	0.36454 (10)	0.38121 (10)	0.0134 (2)	
O13	0.05039 (12)	1.18680 (8)	−0.06791 (8)	0.0204 (2)	
N4	0.39073 (15)	0.87378 (10)	0.02195 (10)	0.0175 (2)	
H4A	0.476 (2)	0.8454 (16)	0.0006 (15)	0.029 (5)*	
H4B	0.340 (2)	0.8382 (16)	0.0802 (16)	0.028 (5)*	
N5	0.19415 (13)	1.02123 (9)	−0.00184 (9)	0.0156 (2)	
H5A	0.140 (2)	0.9944 (16)	0.0570 (16)	0.032 (5)*	
N6	0.39959 (13)	1.03426 (9)	−0.12569 (9)	0.0156 (2)	
C22	0.30056 (16)	1.13631 (11)	−0.16289 (11)	0.0172 (3)	
H22A	0.350 (2)	1.1955 (15)	−0.1755 (14)	0.024 (4)*	
H22B	0.2690 (19)	1.1442 (13)	−0.2225 (13)	0.015 (4)*	
C23	0.54951 (16)	1.00844 (13)	−0.18301 (11)	0.0193 (3)	
H23A	0.540 (3)	0.9830 (19)	−0.2282 (19)	0.051 (7)*	
H23B	0.617 (3)	0.959 (2)	−0.1406 (19)	0.055 (7)*	
H23C	0.599 (3)	1.070 (2)	−0.217 (2)	0.065 (8)*	
C24	0.33392 (15)	0.97084 (11)	−0.03381 (10)	0.0145 (2)	
C25	0.16432 (16)	1.12269 (11)	−0.07475 (10)	0.0161 (3)	
O9	0.22431 (12)	0.75720 (8)	0.21745 (8)	0.0222 (2)	
O10	0.16856 (11)	0.60735 (8)	0.35557 (7)	0.0185 (2)	
H10A	0.080 (3)	0.6511 (19)	0.3638 (17)	0.045 (6)*	
O11	0.69061 (11)	0.75755 (8)	−0.01882 (8)	0.0204 (2)	
O12	0.86113 (11)	0.60775 (8)	0.00786 (8)	0.0185 (2)	
H12A	0.915 (3)	0.649 (2)	−0.0534 (19)	0.051 (7)*	
N3	0.50100 (13)	0.65317 (9)	0.16037 (9)	0.0143 (2)	
C15	0.25904 (15)	0.66364 (11)	0.27266 (10)	0.0158 (3)	
C16	0.41037 (15)	0.59999 (11)	0.24827 (10)	0.0149 (3)	
C17	0.44864 (16)	0.49323 (11)	0.31185 (10)	0.0167 (3)	
H17	0.381 (2)	0.4595 (15)	0.3761 (14)	0.024 (4)*	
C18	0.58702 (16)	0.43822 (11)	0.28135 (11)	0.0181 (3)	
H18	0.613 (2)	0.3676 (16)	0.3202 (14)	0.023 (4)*	
C19	0.68190 (16)	0.49118 (11)	0.18923 (11)	0.0167 (3)	
H19	0.777 (2)	0.4580 (14)	0.1653 (13)	0.021 (4)*	
C20	0.63432 (15)	0.59888 (11)	0.13218 (10)	0.0145 (2)	
C21	0.73088 (15)	0.66371 (11)	0.03255 (10)	0.0152 (3)	
O14	−0.28792 (14)	0.61850 (10)	0.36097 (10)	0.0268 (2)	
H14A	−0.377 (3)	0.6268 (19)	0.3838 (18)	0.048 (7)*	
H14B	−0.258 (3)	0.553 (2)	0.3801 (17)	0.041 (6)*	
O15	−0.08716 (12)	0.72137 (9)	0.38192 (8)	0.0220 (2)	
H15A	−0.160 (3)	0.6886 (18)	0.3832 (17)	0.040 (6)*	
H15B	−0.098 (3)	0.7271 (18)	0.4335 (18)	0.039 (6)*	
O16	0.00515 (12)	−0.05810 (9)	0.18249 (8)	0.0188 (2)	
H16A	0.008 (3)	−0.0312 (19)	0.2190 (18)	0.041 (6)*	
H16B	0.043 (3)	−0.122 (2)	0.2076 (18)	0.049 (7)*	
O17	−0.02251 (12)	0.28295 (9)	0.16386 (8)	0.0189 (2)	
H17A	−0.076 (3)	0.2496 (18)	0.1493 (16)	0.039 (6)*	
H17B	0.029 (3)	0.2388 (19)	0.2012 (18)	0.041 (6)*	
O18	0.59381 (13)	0.31757 (10)	0.57788 (9)	0.0256 (2)	
H18A	0.571 (3)	0.2635 (19)	0.5733 (16)	0.040 (6)*	
H18B	0.673 (3)	0.3345 (19)	0.5322 (19)	0.048 (7)*	
O19	0.81371 (13)	0.17421 (9)	0.11655 (9)	0.0220 (2)	
H19A	0.731 (3)	0.215 (2)	0.1020 (18)	0.050 (7)*	
H19B	0.867 (3)	0.1690 (18)	0.0646 (18)	0.043 (6)*	

### Atomic displacement parameters (Å^2^)

**Table:**

	*U*^11^	*U*^22^	*U*^33^	*U*^12^	*U*^13^	*U*^23^
Cr1	0.01120 (10)	0.00907 (10)	0.00929 (10)	−0.00114 (7)	−0.00217 (7)	−0.00125 (8)
O1	0.0153 (4)	0.0122 (4)	0.0137 (4)	−0.0024 (3)	−0.0039 (3)	−0.0029 (4)
O2	0.0175 (5)	0.0198 (5)	0.0164 (5)	−0.0028 (4)	−0.0066 (4)	−0.0059 (4)
O3	0.0158 (4)	0.0152 (4)	0.0136 (4)	−0.0018 (3)	−0.0041 (3)	−0.0045 (4)
O4	0.0214 (5)	0.0206 (5)	0.0232 (5)	−0.0033 (4)	−0.0063 (4)	−0.0118 (4)
O5	0.0134 (4)	0.0126 (4)	0.0127 (4)	−0.0005 (3)	−0.0017 (3)	−0.0016 (4)
O6	0.0162 (5)	0.0165 (5)	0.0146 (5)	−0.0017 (4)	0.0017 (4)	−0.0023 (4)
O7	0.0140 (4)	0.0125 (4)	0.0125 (4)	−0.0016 (3)	−0.0020 (3)	−0.0026 (4)
O8	0.0144 (5)	0.0185 (5)	0.0191 (5)	0.0005 (4)	−0.0003 (4)	−0.0055 (4)
N1	0.0101 (5)	0.0106 (5)	0.0120 (5)	−0.0004 (4)	−0.0012 (4)	−0.0034 (4)
N2	0.0126 (5)	0.0112 (5)	0.0116 (5)	−0.0019 (4)	−0.0032 (4)	−0.0030 (4)
C1	0.0110 (6)	0.0134 (6)	0.0124 (6)	−0.0004 (5)	−0.0004 (4)	−0.0044 (5)
C2	0.0092 (5)	0.0133 (6)	0.0131 (6)	0.0004 (4)	−0.0014 (4)	−0.0043 (5)
C3	0.0130 (6)	0.0161 (6)	0.0128 (6)	0.0011 (5)	−0.0018 (5)	−0.0029 (5)
C4	0.0145 (6)	0.0107 (6)	0.0163 (6)	0.0001 (5)	0.0008 (5)	−0.0003 (5)
C5	0.0120 (6)	0.0117 (6)	0.0204 (7)	−0.0015 (5)	0.0006 (5)	−0.0057 (5)
C6	0.0097 (5)	0.0131 (6)	0.0154 (6)	−0.0002 (4)	−0.0004 (5)	−0.0061 (5)
C7	0.0109 (6)	0.0161 (6)	0.0144 (6)	0.0006 (5)	−0.0007 (5)	−0.0072 (5)
C8	0.0137 (6)	0.0120 (6)	0.0134 (6)	−0.0020 (5)	−0.0040 (5)	−0.0029 (5)
C9	0.0123 (6)	0.0132 (6)	0.0126 (6)	−0.0029 (5)	−0.0030 (5)	−0.0036 (5)
C10	0.0161 (6)	0.0142 (6)	0.0129 (6)	−0.0037 (5)	−0.0024 (5)	−0.0017 (5)
C11	0.0194 (7)	0.0105 (6)	0.0160 (6)	−0.0018 (5)	−0.0064 (5)	−0.0002 (5)
C12	0.0151 (6)	0.0132 (6)	0.0170 (6)	−0.0002 (5)	−0.0049 (5)	−0.0050 (5)
C13	0.0124 (6)	0.0134 (6)	0.0137 (6)	−0.0023 (5)	−0.0034 (5)	−0.0056 (5)
C14	0.0143 (6)	0.0134 (6)	0.0133 (6)	−0.0031 (5)	−0.0030 (5)	−0.0050 (5)
O13	0.0199 (5)	0.0185 (5)	0.0230 (5)	0.0060 (4)	−0.0071 (4)	−0.0104 (4)
N4	0.0171 (6)	0.0156 (6)	0.0160 (6)	0.0020 (5)	−0.0035 (5)	−0.0040 (5)
N5	0.0153 (5)	0.0152 (5)	0.0145 (6)	0.0010 (4)	−0.0026 (4)	−0.0054 (5)
N6	0.0154 (5)	0.0135 (5)	0.0152 (5)	0.0013 (4)	−0.0033 (4)	−0.0041 (4)
C22	0.0189 (7)	0.0134 (6)	0.0171 (6)	0.0017 (5)	−0.0046 (5)	−0.0046 (5)
C23	0.0163 (7)	0.0232 (7)	0.0163 (7)	0.0009 (5)	−0.0020 (5)	−0.0078 (6)
C24	0.0153 (6)	0.0153 (6)	0.0149 (6)	−0.0005 (5)	−0.0052 (5)	−0.0071 (5)
C25	0.0187 (6)	0.0158 (6)	0.0168 (6)	−0.0008 (5)	−0.0065 (5)	−0.0078 (5)
O9	0.0191 (5)	0.0192 (5)	0.0199 (5)	0.0038 (4)	−0.0009 (4)	−0.0042 (4)
O10	0.0149 (5)	0.0199 (5)	0.0187 (5)	−0.0026 (4)	0.0000 (4)	−0.0071 (4)
O11	0.0169 (5)	0.0182 (5)	0.0198 (5)	0.0005 (4)	−0.0032 (4)	−0.0027 (4)
O12	0.0156 (5)	0.0191 (5)	0.0175 (5)	0.0007 (4)	−0.0008 (4)	−0.0066 (4)
N3	0.0138 (5)	0.0145 (5)	0.0154 (5)	−0.0008 (4)	−0.0040 (4)	−0.0062 (4)
C15	0.0154 (6)	0.0177 (6)	0.0151 (6)	−0.0021 (5)	−0.0036 (5)	−0.0067 (5)
C16	0.0157 (6)	0.0154 (6)	0.0156 (6)	−0.0021 (5)	−0.0044 (5)	−0.0070 (5)
C17	0.0184 (6)	0.0154 (6)	0.0163 (6)	−0.0041 (5)	−0.0037 (5)	−0.0050 (5)
C18	0.0209 (7)	0.0124 (6)	0.0200 (7)	−0.0008 (5)	−0.0073 (5)	−0.0038 (5)
C19	0.0156 (6)	0.0155 (6)	0.0206 (7)	0.0006 (5)	−0.0057 (5)	−0.0084 (5)
C20	0.0148 (6)	0.0151 (6)	0.0155 (6)	−0.0017 (5)	−0.0048 (5)	−0.0066 (5)
C21	0.0139 (6)	0.0178 (6)	0.0161 (6)	−0.0014 (5)	−0.0046 (5)	−0.0077 (5)
O14	0.0180 (6)	0.0181 (6)	0.0398 (7)	0.0000 (4)	−0.0020 (5)	−0.0100 (5)
O15	0.0191 (5)	0.0293 (6)	0.0188 (5)	−0.0005 (4)	−0.0025 (4)	−0.0122 (5)
O16	0.0224 (5)	0.0170 (5)	0.0175 (5)	0.0006 (4)	−0.0048 (4)	−0.0080 (4)
O17	0.0174 (5)	0.0201 (5)	0.0166 (5)	0.0003 (4)	−0.0054 (4)	−0.0044 (4)
O18	0.0191 (5)	0.0295 (6)	0.0342 (6)	−0.0083 (4)	0.0033 (5)	−0.0204 (5)
O19	0.0187 (5)	0.0239 (5)	0.0215 (5)	0.0022 (4)	−0.0059 (4)	−0.0080 (4)

### Geometric parameters (Å, °)

**Table:**

Cr1—N1	1.9733 (11)	N5—C25	1.3782 (17)
Cr1—N2	1.9769 (11)	N5—H5A	0.85 (2)
Cr1—O5	1.9842 (9)	N6—C24	1.3259 (17)
Cr1—O3	1.9942 (10)	N6—C23	1.4581 (18)
Cr1—O1	1.9947 (9)	N6—C22	1.4616 (17)
Cr1—O7	1.9974 (9)	C22—C25	1.5171 (19)
O1—C1	1.3009 (15)	C22—H22A	0.938 (19)
O2—C1	1.2262 (16)	C22—H22B	0.952 (17)
O3—C7	1.3106 (16)	C23—H23A	0.90 (3)
O4—C7	1.2176 (17)	C23—H23B	0.95 (3)
O5—C8	1.2975 (15)	C23—H23C	0.92 (3)
O6—C8	1.2283 (16)	O9—C15	1.2204 (17)
O7—C14	1.3039 (16)	O10—C15	1.3107 (17)
O8—C14	1.2244 (16)	O10—H10A	0.91 (2)
N1—C6	1.3329 (17)	O11—C21	1.2184 (17)
N1—C2	1.3366 (17)	O12—C21	1.3157 (16)
N2—C13	1.3365 (17)	O12—H12A	0.91 (2)
N2—C9	1.3373 (16)	N3—C20	1.3390 (17)
C1—C2	1.5160 (18)	N3—C16	1.3391 (17)
C2—C3	1.3861 (18)	C15—C16	1.5031 (18)
C3—C4	1.394 (2)	C16—C17	1.3926 (19)
C3—H3	0.920 (17)	C17—C18	1.386 (2)
C4—C5	1.395 (2)	C17—H17	0.970 (18)
C4—H4	0.932 (17)	C18—C19	1.393 (2)
C5—C6	1.3886 (18)	C18—H18	0.909 (19)
C5—H5	0.934 (18)	C19—C20	1.3942 (19)
C6—C7	1.5145 (18)	C19—H19	0.935 (18)
C8—C9	1.5172 (18)	C20—C21	1.5099 (19)
C9—C10	1.3859 (18)	O14—H14A	0.79 (3)
C10—C11	1.3949 (19)	O14—H14B	0.83 (2)
C10—H10	0.927 (19)	O15—H15A	0.86 (2)
C11—C12	1.3965 (19)	O15—H15B	0.77 (2)
C11—H11	0.944 (18)	O16—H16A	0.78 (2)
C12—C13	1.3858 (18)	O16—H16B	0.83 (3)
C12—H12	0.942 (17)	O17—H17A	0.85 (2)
C13—C14	1.5161 (18)	O17—H17B	0.79 (2)
O13—C25	1.2149 (17)	O18—H18A	0.84 (2)
N4—C24	1.3117 (18)	O18—H18B	0.84 (3)
N4—H4A	0.83 (2)	O19—H19A	0.85 (3)
N4—H4B	0.85 (2)	O19—H19B	0.81 (2)
N5—C24	1.3731 (17)		
			
N1—Cr1—N2	172.88 (4)	N2—C13—C14	110.86 (11)
N1—Cr1—O5	106.16 (4)	C12—C13—C14	128.84 (12)
N2—Cr1—O5	79.39 (4)	O8—C14—O7	124.76 (12)
N1—Cr1—O3	78.84 (4)	O8—C14—C13	121.81 (12)
N2—Cr1—O3	96.78 (4)	O7—C14—C13	113.42 (11)
O5—Cr1—O3	91.39 (4)	C24—N4—H4A	120.6 (14)
N1—Cr1—O1	79.13 (4)	C24—N4—H4B	120.3 (13)
N2—Cr1—O1	105.30 (4)	H4A—N4—H4B	119.1 (19)
O5—Cr1—O1	93.27 (4)	C24—N5—C25	110.51 (11)
O3—Cr1—O1	157.91 (4)	C24—N5—H5A	123.0 (14)
N1—Cr1—O7	96.08 (4)	C25—N5—H5A	126.3 (14)
N2—Cr1—O7	78.42 (4)	C24—N6—C23	125.88 (12)
O5—Cr1—O7	157.76 (4)	C24—N6—C22	110.40 (11)
O3—Cr1—O7	92.80 (4)	C23—N6—C22	123.72 (11)
O1—Cr1—O7	91.01 (4)	N6—C22—C25	102.34 (11)
C1—O1—Cr1	117.31 (8)	N6—C22—H22A	110.6 (11)
C7—O3—Cr1	117.92 (8)	C25—C22—H22A	111.4 (11)
C8—O5—Cr1	117.46 (8)	N6—C22—H22B	111.1 (10)
C14—O7—Cr1	117.96 (8)	C25—C22—H22B	110.6 (10)
C6—N1—C2	123.01 (11)	H22A—C22—H22B	110.6 (15)
C6—N1—Cr1	118.48 (9)	N6—C23—H23A	111.2 (15)
C2—N1—Cr1	118.06 (9)	N6—C23—H23B	112.8 (15)
C13—N2—C9	123.11 (11)	H23A—C23—H23B	110 (2)
C13—N2—Cr1	118.99 (9)	N6—C23—H23C	109.5 (17)
C9—N2—Cr1	117.84 (9)	H23A—C23—H23C	109 (2)
O2—C1—O1	126.04 (12)	H23B—C23—H23C	104 (2)
O2—C1—C2	120.05 (11)	N4—C24—N6	126.64 (13)
O1—C1—C2	113.91 (11)	N4—C24—N5	122.86 (13)
N1—C2—C3	120.18 (12)	N6—C24—N5	110.49 (11)
N1—C2—C1	111.06 (11)	O13—C25—N5	125.75 (13)
C3—C2—C1	128.76 (12)	O13—C25—C22	128.00 (13)
C2—C3—C4	117.72 (12)	N5—C25—C22	106.25 (11)
C2—C3—H3	119.9 (11)	C15—O10—H10A	107.6 (14)
C4—C3—H3	122.4 (10)	C21—O12—H12A	110.0 (15)
C3—C4—C5	121.20 (12)	C20—N3—C16	117.38 (11)
C3—C4—H4	120.1 (10)	O9—C15—O10	124.43 (13)
C5—C4—H4	118.7 (11)	O9—C15—C16	122.17 (12)
C6—C5—C4	117.61 (12)	O10—C15—C16	113.34 (12)
C6—C5—H5	118.9 (11)	N3—C16—C17	123.71 (12)
C4—C5—H5	123.5 (11)	N3—C16—C15	114.63 (11)
N1—C6—C5	120.24 (12)	C17—C16—C15	121.64 (12)
N1—C6—C7	111.66 (11)	C18—C17—C16	118.11 (13)
C5—C6—C7	128.04 (12)	C18—C17—H17	121.6 (11)
O4—C7—O3	125.86 (12)	C16—C17—H17	120.3 (11)
O4—C7—C6	121.13 (12)	C17—C18—C19	119.22 (13)
O3—C7—C6	112.99 (11)	C17—C18—H18	119.9 (12)
O6—C8—O5	125.49 (12)	C19—C18—H18	120.8 (12)
O6—C8—C9	120.42 (11)	C18—C19—C20	118.15 (13)
O5—C8—C9	114.09 (11)	C18—C19—H19	122.6 (11)
N2—C9—C10	119.86 (12)	C20—C19—H19	119.2 (11)
N2—C9—C8	111.14 (11)	N3—C20—C19	123.42 (12)
C10—C9—C8	129.00 (12)	N3—C20—C21	114.59 (11)
C9—C10—C11	118.03 (12)	C19—C20—C21	122.00 (12)
C9—C10—H10	122.6 (11)	O11—C21—O12	124.35 (13)
C11—C10—H10	119.4 (11)	O11—C21—C20	122.94 (12)
C10—C11—C12	121.08 (12)	O12—C21—C20	112.71 (11)
C10—C11—H11	118.9 (11)	H14A—O14—H14B	109 (2)
C12—C11—H11	120.0 (11)	H15A—O15—H15B	110 (2)
C13—C12—C11	117.60 (12)	H16A—O16—H16B	106 (2)
C13—C12—H12	120.7 (10)	H17A—O17—H17B	108 (2)
C11—C12—H12	121.7 (10)	H18A—O18—H18B	104 (2)
N2—C13—C12	120.29 (12)	H19A—O19—H19B	108 (2)
			
N1—Cr1—O1—C1	−2.46 (9)	C5—C6—C7—O4	−4.4 (2)
N2—Cr1—O1—C1	171.81 (9)	N1—C6—C7—O3	−2.96 (15)
O5—Cr1—O1—C1	−108.30 (9)	C5—C6—C7—O3	174.13 (12)
O3—Cr1—O1—C1	−6.46 (16)	Cr1—O5—C8—O6	−176.91 (10)
O7—Cr1—O1—C1	93.54 (9)	Cr1—O5—C8—C9	2.87 (14)
N1—Cr1—O3—C7	0.88 (9)	C13—N2—C9—C10	1.76 (19)
N2—Cr1—O3—C7	−173.43 (9)	Cr1—N2—C9—C10	179.04 (10)
O5—Cr1—O3—C7	107.08 (9)	C13—N2—C9—C8	−177.99 (11)
O1—Cr1—O3—C7	4.89 (16)	Cr1—N2—C9—C8	−0.71 (14)
O7—Cr1—O3—C7	−94.76 (9)	O6—C8—C9—N2	178.40 (12)
N1—Cr1—O5—C8	172.87 (9)	O5—C8—C9—N2	−1.39 (16)
N2—Cr1—O5—C8	−2.55 (9)	O6—C8—C9—C10	−1.3 (2)
O3—Cr1—O5—C8	94.09 (9)	O5—C8—C9—C10	178.89 (13)
O1—Cr1—O5—C8	−107.51 (9)	N2—C9—C10—C11	−0.54 (19)
O7—Cr1—O5—C8	−6.78 (16)	C8—C9—C10—C11	179.17 (12)
N1—Cr1—O7—C14	−170.15 (9)	C9—C10—C11—C12	−0.8 (2)
N2—Cr1—O7—C14	5.26 (9)	C10—C11—C12—C13	0.9 (2)
O5—Cr1—O7—C14	9.50 (16)	C9—N2—C13—C12	−1.60 (19)
O3—Cr1—O7—C14	−91.09 (9)	Cr1—N2—C13—C12	−178.85 (9)
O1—Cr1—O7—C14	110.67 (9)	C9—N2—C13—C14	177.15 (11)
O5—Cr1—N1—C6	−90.96 (10)	Cr1—N2—C13—C14	−0.10 (14)
O3—Cr1—N1—C6	−2.76 (9)	C11—C12—C13—N2	0.21 (19)
O1—Cr1—N1—C6	178.77 (10)	C11—C12—C13—C14	−178.29 (12)
O7—Cr1—N1—C6	88.91 (10)	Cr1—O7—C14—O8	172.16 (10)
O5—Cr1—N1—C2	96.49 (9)	Cr1—O7—C14—C13	−6.72 (14)
O3—Cr1—N1—C2	−175.31 (10)	N2—C13—C14—O8	−174.56 (12)
O1—Cr1—N1—C2	6.22 (9)	C12—C13—C14—O8	4.0 (2)
O7—Cr1—N1—C2	−83.64 (9)	N2—C13—C14—O7	4.35 (15)
O5—Cr1—N2—C13	179.09 (10)	C12—C13—C14—O7	−177.04 (13)
O3—Cr1—N2—C13	88.95 (10)	C24—N6—C22—C25	1.04 (14)
O1—Cr1—N2—C13	−90.40 (10)	C23—N6—C22—C25	−178.54 (12)
O7—Cr1—N2—C13	−2.54 (9)	C23—N6—C24—N4	−0.3 (2)
O5—Cr1—N2—C9	1.70 (9)	C22—N6—C24—N4	−179.89 (13)
O3—Cr1—N2—C9	−88.45 (9)	C23—N6—C24—N5	178.95 (12)
O1—Cr1—N2—C9	92.21 (9)	C22—N6—C24—N5	−0.61 (15)
O7—Cr1—N2—C9	−179.93 (10)	C25—N5—C24—N4	179.15 (12)
Cr1—O1—C1—O2	177.87 (10)	C25—N5—C24—N6	−0.15 (16)
Cr1—O1—C1—C2	−1.13 (14)	C24—N5—C25—O13	−179.32 (13)
C6—N1—C2—C3	−0.63 (19)	C24—N5—C25—C22	0.81 (15)
Cr1—N1—C2—C3	171.56 (9)	N6—C22—C25—O13	179.04 (13)
C6—N1—C2—C1	179.57 (11)	N6—C22—C25—N5	−1.09 (14)
Cr1—N1—C2—C1	−8.24 (13)	C20—N3—C16—C17	1.25 (19)
O2—C1—C2—N1	−173.12 (11)	C20—N3—C16—C15	−176.95 (11)
O1—C1—C2—N1	5.95 (15)	O9—C15—C16—N3	−1.70 (19)
O2—C1—C2—C3	7.1 (2)	O10—C15—C16—N3	175.76 (11)
O1—C1—C2—C3	−173.83 (12)	O9—C15—C16—C17	−179.95 (13)
N1—C2—C3—C4	1.51 (19)	O10—C15—C16—C17	−2.48 (18)
C1—C2—C3—C4	−178.72 (12)	N3—C16—C17—C18	−1.4 (2)
C2—C3—C4—C5	−0.50 (19)	C15—C16—C17—C18	176.71 (12)
C3—C4—C5—C6	−1.35 (19)	C16—C17—C18—C19	0.1 (2)
C2—N1—C6—C5	−1.34 (19)	C17—C18—C19—C20	1.2 (2)
Cr1—N1—C6—C5	−173.49 (9)	C16—N3—C20—C19	0.16 (19)
C2—N1—C6—C7	176.01 (11)	C16—N3—C20—C21	−179.88 (11)
Cr1—N1—C6—C7	3.86 (14)	C18—C19—C20—N3	−1.4 (2)
C4—C5—C6—N1	2.27 (19)	C18—C19—C20—C21	178.67 (12)
C4—C5—C6—C7	−174.60 (12)	N3—C20—C21—O11	−0.66 (19)
Cr1—O3—C7—O4	179.27 (10)	C19—C20—C21—O11	179.30 (13)
Cr1—O3—C7—C6	0.84 (13)	N3—C20—C21—O12	179.64 (11)
N1—C6—C7—O4	178.53 (12)	C19—C20—C21—O12	−0.40 (18)

### Hydrogen-bond geometry (Å, °)

**Table:**

*D*—H···*A*	*D*—H	H···*A*	*D*···*A*	*D*—H···*A*
N4—H4A···O11	0.83 (2)	2.08 (2)	2.8934 (16)	167.3 (19)
N4—H4B···O9	0.85 (2)	1.98 (2)	2.8343 (16)	178.5 (8)
O10—H10A···O15	0.91 (2)	1.63 (2)	2.5382 (15)	176 (2)
O14—H14B···O8	0.83 (2)	2.01 (2)	2.8255 (16)	169 (2)
O15—H15A···O14	0.86 (2)	1.82 (2)	2.6718 (17)	168 (2)
O16—H16A···O4	0.78 (2)	2.07 (2)	2.8006 (15)	156 (2)
O17—H17B···O3	0.79 (2)	1.99 (2)	2.7758 (14)	172 (2)
O18—H18A···O2	0.84 (2)	1.98 (2)	2.7927 (15)	162 (2)
O19—H19A···O6	0.85 (3)	1.95 (3)	2.7763 (15)	164 (2)
N5—H5A···O16^i^	0.85 (2)	1.87 (2)	2.7175 (16)	175 (2)
O12—H12A···O17^ii^	0.91 (2)	1.66 (3)	2.5720 (14)	176 (2)
O14—H14A···O18^iii^	0.79 (3)	1.98 (3)	2.7546 (17)	167 (2)
O15—H15B···O7^iii^	0.77 (2)	2.16 (2)	2.9105 (15)	162 (2)
O16—H16B···O9^iv^	0.83 (3)	2.05 (3)	2.8083 (15)	152 (2)
O17—H17A···O19^v^	0.85 (2)	1.84 (2)	2.6916 (16)	178 (2)
O18—H18B···O8^vi^	0.84 (3)	2.12 (3)	2.9558 (15)	171 (2)
O19—H19B···O13^vii^	0.81 (2)	2.17 (2)	2.9531 (16)	162 (2)
C5—H5···O7^viii^	0.94 (2)	2.392 (19)	3.251 (2)	152.2 (15)
C10—H10···O6^ii^	0.929 (19)	2.283 (19)	3.0919 (17)	145.4 (18)

Symmetry codes: (i) *x*, *y*+1, *z*; (ii) −*x*+1, −*y*+1, −*z*; (iii) −*x*, −*y*+1, −*z*+1; (iv) *x*, *y*−1, *z*; (v) *x*−1, *y*, *z*; (vi) *x*+1, *y*, *z*; (vii) *x*+1, *y*−1, *z*; (viii) −*x*, −*y*, −*z*+1.

## References

[bb1] Aghabozorg, H., Manteghi, F. & Sheshmani, S. (2008). *J. Iran. Chem. Soc.* **5**, 184–227.

[bb2] Aghabozorg, H., Ramezanipour, F., Soleimannejad, J., Sharif, M. A., Shokrollahi, A., Shamsipur, M., Moghimi, A., Attar Gharamaleki, J., Lippolis, V. & Blake, A. J. (2008). *Polish J* *Chem.* **82**, 487–507.

[bb3] Bruker (2007). *APEX2* and *SAINT* Bruker AXS Inc., Madison, Wisconsin, USA.

[bb4] Moghimi, A., Sharif, M. A. & Aghabozorg, H. (2004). *Acta Cryst.* E**60**, o1790–o1792.

[bb5] Moghimi, A., Sharif, M. A., Shokrollahi, A., Shamsipur, M. & Aghabozorg, H. (2005). *Z. Anorg. Allg. Chem.* **631**, 902–908.

[bb6] Sheldrick, G. M. (1996). *SADABS* University of Göttingen, Germany.

[bb7] Sheldrick, G. M. (2008). *Acta Cryst.* A**64**, 112–122. 10.1107/S0108767307043930 18156677

